# Interrelationships between Skin Structure, Function, and Microbiome of Pregnant Females and Their Newborns: Study Protocol for a Prospective Cohort Study

**DOI:** 10.1155/2021/4163705

**Published:** 2021-11-17

**Authors:** Doris Wilborn, Jan Kottner, Kathrin Hillmann, Sa Xu, Frank Konietschke, Ulrike Blume-Peytavi

**Affiliations:** ^1^Clinical Research Center for Hair and Skin Science, Department of Dermatology, Venereology and Allergology, Charité–Universitätsmedizin Berlin, Charitéplatz 1, Berlin 10117, Germany; ^2^Institute of Clinical Nursing Science, Charité–Universitätsmedizin Berlin, Charitéplatz 1, Berlin 10117, Germany; ^3^Dehaa Rossun Research Center, Kowloon, Hongkong, China; ^4^Department of Biometry and Clinical Epidemiology, Charité–Universitätsmedizin Berlin, Charitéplatz 1, Berlin 10117, Germany

## Abstract

**Background:**

Pregnancy leads to several skin changes, but evidence about structural and functional skin changes is scarce. Findings on skin structure and function in children in their first year reveal rapid skin maturation, but evidence indicates that in particular, water holding and transport mechanisms are different from adults. Important questions include whether maternal cutaneous properties predict infant skin condition, and if so, how. This is especially relevant for the skin's microbiome because it closely interacts with the host and is assumed to play a role in many skin diseases. Therefore, the study objective is to explore characteristics of skin and hair of pregnant women and their newborns during pregnancy and in the first six months after delivery and their associations.

**Methods:**

The study has an observational longitudinal design. We are recruiting pregnant females between 18 and 45 years using advertisement campaigns in waiting areas of gynecologists and hospital's outpatient services. A final sample size of *n* = 100 women is the target. We perform noninvasive, standardized skin, hair, and skin microbiome measurements. We establish the baseline visit during pregnancy until at the latest four weeks before delivery. We schedule follow-up visits four weeks and six months after birth for mothers and their newborns. We will calculate descriptive statistical methods using frequencies and associations over time depending on scale levels of the measurements. *Discussion*. The majority of previous studies that have investigated infants' skin microbiome and its associations used cross-sectional designs and focused on selected characteristics in small samples. In our longitudinal study, we will characterize a broad range of individual and environmental characteristics of mothers and their newborns to evaluate interrelationships with skin parameters and their changes over time. Considering the combination of these multiple variables and levels will allow for a deeper understanding of the complex interrelationship of the newborn's skin maturation. This trial is registered with ClinicalTrials.gov (Identifier: NCT04759924).

## 1. Background

The skin is widely considered the largest organ of the human body, and it fulfills a variety of essential functions. One of the most important functions is protection. However, there are certain periods in life when skin health is challenged [1]. Two of these periods are during pregnancy for women and during the first few months after birth for both mothers and their newborns. It is well known that pregnant women may undergo a wide range of skin and hair changes such as pigmentary, vascular, hair growth, nail, and connective tissue alterations [2–6]. These effects are also assumed to continue after childbirth [7]. The scalp hair is particularly affected: hormonal changes during pregnancy lead to a high anagen rate of nearly 90% of all hair follicles during the second and third quarters of pregnancy [8]. However, some women experience postpartum diffuse hair loss, which is enhanced in women already having hair loss problems prior to pregnancy. This hair growth disorder is known as telogen effluvium, lasting between six weeks and up to six months after delivery [9]. But why there are significant individual differences in postnatal hair loss remains unclear. So detailed evidence about the structure and function of skin and hair changes during and after pregnancy is largely lacking.

In contrast, evidence about skin function and skin structure in newborns and infants is more common. Immediately after birth, the rapid skin barrier maturation is well studied [10], but evidence indicates that in particular, the water holding and transport mechanisms are very different to adults in the first year of life [11]. Transepidermal water loss (TEWL) measurements indicate that after the first month after birth, TEWL seems to become similar to the adult skin [10, 12, 13]. Until one year of age, TEWL increases [10, 14, 15], and pH values of the skin surface decline from immediately after birth to several months later, indicating skin barrier maturation [10, 15, 16].

In recent years, there has been an increasing interest in associations of the skin microbiome and skin health [17]. The above-described physiological changes of the skin from birth during the first year of life may also interact with the skin microbiome [18]. Oranges et al. conclude that the skin microbiome of newborns resembles the microbiome of moist skin sites in adults [13]. Other study results indicate associations between the richness and diversity of the microbiome, the skin, and environmental properties [18–21]. This signifies complex interrelationships between physiological development, microbial colonization and growth, and external influences.

Until now, few studies have explored possible associations of skin function and microbiome between mothers and newborns. Dominguez-Bello et al. showed that the method of delivery (vaginal or via C-section) is linked to the diversity of the cutaneous bacterial colonization [19]. Additionally, results of 50 mother-child pairs indicate similarities of the children's bacterial genera to those of their own mothers [22]. Other study results suggest associations between environmental conditions, age, and the skin microbiome [23, 24].

However, most study results are based on cross-sectional study designs, ignoring changes over time. Because of the multiple interactions between skin structure, function, and microbiome, it is necessary to consider and measure all aspects over time.

## 2. Methods/Design

### 2.1. Aim

The aim is to measure skin and hair characteristics of mothers and newborns over time and determine demographic, environmental, and health characteristics associated with skin microbiome development.

Therefore, we will answer the following research questions:

1. Are there associations between health, skin, and hair characteristics of mothers and newborns?
2. How does the skin microbiome change during pregnancy and after birth?
3. Do maternal health and skin characteristics predict the skin characteristics of newborns and infants?

### 2.2. Study Design

We perform a longitudinal, descriptive cohort study including females during pregnancy and puerperium, and their newborns until six months of age shown in Table 1.

### 2.3. Setting

We invite women after the first pregnancy trimester from the federal state of Berlin (Germany) during their visits to gynecologists and/or midwives. We conduct inclusion and all study visits and measurement procedures at the Clinical Research Center for Hair and Skin Science at the Department of Dermatology at the Charité–Universitätsmedizin Berlin (Germany). If they meet the eligibility criteria, women are included in the study. Upon inclusion in the study after the baseline visit, we follow up with the women and their newborns four weeks after delivery and again six months after delivery.

### 2.4. Participants

Participants are pregnant females between 18 and 45 years old and residing in Berlin (Germany). Prerequisites for study inclusion are individual written informed consent, clinically healthy skin and hair appearance, as well as being free of any dermatological condition. Skin afflictions, which may interfere with the study assessments, especially atopic dermatitis, scars, or other lesions at the investigational site result in exclusion. Women must be of general good and stable health as confirmed by medical history and by a physical examination. They must accept to abstain from sunbathing and solariums and to agree to stay consistent in using their individual skin cleaning and caring routines during the study.

Women are excluded due to regular smoking, regular alcohol intake, or criteria related to diseases such as any dermatological condition or skin affliction, which may interfere with the study assessments. These could be any inflammatory dermatosis (psoriasis, atopic dermatitis, or other lesions at the investigational sites) or clinically significant, possibly unstable medical conditions due to pregnancy such as gestosis, eclampsia, or thrombosis. Criteria related to treatments and products such as current topical or systemic treatment possibly affecting the skin (diuretics, cholesterol-lowering drugs, and hormones) during the past four weeks are further exclusion criteria. Additionally, therapeutic ultraviolet radiation or increased ultraviolet exposure within six weeks before inclusion leads to exclusion. For safety, other medical reasons, and any individual reasons, study participation can be stopped.

### 2.5. Variables

Due to the descriptive and exploratory nature of this study, we assess a broad range of characteristics and variables. Skin structure is measured by means of skin surface topography, epidermal thickness, skin stiffness, and skin elasticity. Skin function is measured by means of stratum corneum hydration (SCH), TEWL, and skin surface pH, which are established parameters to characterize the skin barrier function. Skin microbiome is defined as bacterial diversity, and in this study, the relative abundance of phylotypes, (operational taxonomic units, OTUs) will be measured. Hair growth is measured on the central and occipital scalp by means of hair density and hair widths. The definitions of the variables are shown in Table 2 and further explained in the following paragraph.

### 2.6. Data Sources/Measurement

We train all data collectors in accurately obtaining the variables of interest. We use paper source data and electronic case report forms to document all study variables of interest.

#### 2.6.1. Dermatological Examination

The investigator performs the dermatological examination during the visits together with a subject's interview on medical history and treatments to check the subject's eligibility.

#### 2.6.2. Measurements of Skin Physiology

We measure the physiological skin parameters of the females and the newborns at one area of the right inner forearm. All measurement procedures are based on internal standardized operating procedures as part of the quality management system of the study site. Before the assessment measurements, subjects rest for 30 minutes in a room with controlled environmental conditions (22°C ± 2°C room temperature; 50% ± 10% relative humidity) for acclimatization. During acclimatization, the skin of the investigational sites is uncovered and exposed to ambient air. The parameters are measured in the following order: skin swab, TEWL, SCH, pH, Cutometry, OCT, and Visioscan. The order and exact skin areas are arranged in such a way that any measurement procedure will not affect the readings of the subsequent measurement [25].

#### 2.6.3. Skin Surface Topography

Based on the definition of the EEMCO guidance [26], the skin surface topography is measured with “Visioscan VC 98 USB” (Courage + Khazaka Electronics GmbH, Germany). The skin is scanned with a high-resolution camera to measure mean roughness. Mean roughness (Rz) is presented in means (SD) and median (IQR).

#### 2.6.4. Epidermal Thickness

Optical coherence tomography (OCT; THORLABS TELESTO, Spectral Domain OCT System) allows for a noninvasive imaging of the epidermis [27, 28]. Based on the obtained images, epidermal thickness is calculated using internal standardized operational procedures.

#### 2.6.5. Skin Stiffness and Elasticity

The total extensibility of the skin (Uf, mm) is measured using the “Cutometer MPA 580” (Courage + Khazaka Electronics GmbH, Germany) according to guidance by the EECMO group for the assessment of biomechanical skin properties [29].

#### 2.6.6. Transepidermal Water Loss

TEWL (*g* per hour per m^2^) is measured using the “Tewameter TM 300” (Courage + Khazaka, Electronics GmbH, Germany). This is an open chamber method, and the results are compared to published reference values in adults and newborns [10, 30]. Empirical evidence supports the reliability of this estimate at the volar forearm [31].

#### 2.6.7. Skin Surface pH

Skin pH is measured using the “Skin-ph-Meter®PH 905 device” (Courage + Khazaka, Electronics GmbH, Germany). This investigation is highly reliable at the volar forearm [31] when applying the standard operating procedures. Reference values of human skin have been reported to range from 4 to 6 [32, 33].

#### 2.6.8. Stratum Corneum Hydration

SCH is measured by using the “Corneometer CM 825” (Courage + Khazaka, Electronics GmbH, Germany). The measurement is based on the differences in the dielectric constant of water and other substances within the stratum corneum. The arbitrary units for SCH measurements range from 0 to 120, with higher readings indicating higher SCH [34].

#### 2.6.9. Skin Microbiome and Analysis

We follow the analytic test methods put forward by McInnes and Cutting in the “Human microbiome project,” for describing the microbiome distribution of bacterial species in this study [35]. According to the latest recommendations by the involved test laboratory, a swab is taken with aseptic techniques at the volar forearm. The swab is moistened with sterile NaCl 0.9% + 0.5% Tween 20 solution and then rubbed back and forth for 30 seconds. Afterwards, the swab head is inserted into the tube containing DNA/RNA Shield of Zymo Research, Irvine, USA. The head of the swab is then aseptically cut from the handle, and the tube cap is screwed back in place. The skin microbiome is defined as bacterial diversity. The relative abundance of phylotypes (operational taxonomic units, OTUs) is measured by the alpha diversity and Shannon index.

#### 2.6.10. Hair Measurements

Hair measurements are taken on the central and occipital head area in women and on the central head area in newborns/infants. Standardized macro photographs are taken to measure hair growth and structure. We use the Visiomed D200 evo camera of Vectra H1 Hair Metrix, Canfield Scientific, Fairfield, USA. The camera is placed on the selected area of the participant's scalp to take a picture as a noninvasive method. At the selected area (central and occipital), the hair is parted with a comb, preparing the hair region for investigation. The following quantitative hair measurements are calculated automatically by the software system: hair count per cm^2^, sum of hair width per cm^2^, terminal to vellus ratio, and average hair width (*μ*m). Additionally, the hair colour is estimated by a 10-point hair colour scale [36].

### 2.7. Bias

#### 2.7.1. Information Bias

During the study period, the risk of losing participants is addressed with proper information and close support in the beginning and throughout the study. An expense allowance is provided. If a woman is included during the early phase of pregnancy, a phone call is scheduled three months after inclusion or at the latest one week before the due date. This phone call allows us to check the current situation, as well as to check the date for the second visit (t1). Similar to this procedure, the women are called one week before the scheduled third visit (t2). If the women are not reachable, phone calls are repeated twice, and in addition, emails are sent and repeated twice. If there is no response, a letter by registered post is sent. If there is no response after all contact attempts, the woman is then excluded from the study.

In order to estimate the outcomes accurately, all investigations are following internal standardized operating or technical procedures, with staff exclusively allocated to the study performing them.

#### 2.7.2. Selection Bias

Due to the sampling of eligible women restricted to one geographical area, a potential sampling bias is addressed by comparing all relevant characteristics of the included women with national data. Due to the voluntary nature of inclusion in the study, a sampling bias might occur. Therefore, relevant characteristics of the included women are compared to the study population to evaluate potential differences. To address a potential response bias, the age distribution and the number of pregnancies of the included women are considered and compared to national data of the mean age of women at delivery of their first child.

### 2.8. Study Size

Due to the exploratory character of this study, formal sample size calculation is not performed. We aim to include 100 pregnant females and their newborns/infants to obtain means and proportions with an acceptable range of uncertainty. A loss-to-follow-up of one-third is assumed resulting in a total of *n* = 150 mothers to be included.

### 2.9. Statistical Methods

We will describe the demographic characteristics of the subjects, using absolute and relative frequencies, means, and standard deviations (SD). Metric outcomes will be described using means, SD, medians, and interquartile ranges (IQR) per group and time point. Ordinally, scaled outcomes will be described using medians and IQR per group and time point. Associations of skin parameters between mothers and their newborns/infants will be described using correlation coefficients (Kendall's Tau). Linear mixed models (metric analysis), as well as nonparametric rank-based methods for longitudinal data (ordinal data or skewed endpoints), will be used to assess differences between groups over time (and possible interactions). Furthermore, the Munzel test r (nonparametric) or paired *t* tests (metric observations) will be used to compare paired data. Confidence intervals (95%) will be computed for the effects of interest. Due to the explanatory character of the study, all statistical analyses are exploratory, and thus, all *P*-values and confidence intervals are considered descriptive. If missing values occur, we discuss them on an individual level and use all available cases in the analysis. All statistical methods will be performed using validated software.

#### 2.9.1. Methods to Examine Subgroups and Interactions

The following subgroup analyses are planned: according to age groups of the females, comparisons will be made for Q1/Q4-age-related groups. Due to the predisposition for atopic dermatitis (AD), an analysis for existing or non-existing AD will be made. Because of a possible association between mode of delivery and the skin microbiome, comparisons will be made for vaginal and cesarean delivery.

#### 2.9.2. Missing Data Management

Because of the long individual follow-up, the attrition rates are difficult to predict. After the inclusion of *n* = 150 women, we assume the number of dropouts to be *n* = 50. Because this is a single cohort of women and their newborns, missing data will not be dependent on different exposures. However, we will compare the groups of subjects with missing data and groups of subjects with complete data regarding main outcome variables to estimate potential bias. Secondly, we will estimate the influence of main predictor variables, such as age, on the occurrence of missing data. Based on the proportion of missing values and the assumed bias, appropriate multiple imputation methods for categorical and continuous variables will be applied [37].

### 2.10. Dissemination Policy

Subjects or parents/guardians have the right to ask the investigator for a summary of the study results, as soon as they are available. Results are planned to be published in an international peer-reviewed scientific journal. The content and structure of the manuscript will follow the STROBE statement for observational studies [38, 39]. Authorship will follow the criteria of the International Committee of Medical Journal Editors.

## 3. Discussion

Most of the previous studies investigating the infant's skin microbiome and its associations were conducted using cross-sectional designs and focused on selected characteristics in small samples. In our longitudinal study, we are characterizing a broad range of individual and environmental characteristics of mothers and their newborns to evaluate interrelationships with skin parameters and their changes over the period of at least eight months. Because it is well known that cutaneous changes occur rapidly during and after pregnancy and after birth, the chosen times for measurements are likely to capture these changes. Connecting demographic, environmental characteristics, as well as biophysical skin parameters and the skin microbiome has never been done in this setting to date. Considering these multiple variables and levels together will allow for a deeper understanding of the complex interrelationship of the newborn's skin maturation and allow generating hypotheses to be tested in larger confirmatory studies. By analyzing factors influencing the cutaneous microbiome of the newborns, the results can support recommendations for environmental or individual living conditions and may help predict cutaneous risk in newborns and infants based on their mothers.

## 4. Trial Status

The study is based on the study protocol, from 21 October 2020, Version No. 2. The recruitment phase started on 10 March 2021. The approximate month when recruitment will be completed is estimated to be June 2022.

## Figures and Tables

**Table 1:** Timeline for women and newborns.

	Inclusion baseline visit during pregnancy (16^th^–36^th^ week) t0	Delivery + 4 weeks t1	Delivery + 6 months (end of the study) t2

Informed consent	*x*	*x* newborn	
Inclusion/exclusion criteria	*x*	*x* newborn	
Medical examination	*x*	*x* ^1^	*x* ^1^
Dermatological examination	*x*	*x* ^1^	*x* ^1^
Skin measurements	*x*	*x* ^1^	*x* ^1^
Skin microbiome	*x*	*x* ^1^	*x* ^1^
Hair metrix analysis	*x*	*x* ^1^	*x* ^1^

*x* ^1^ = visit for mother and baby.

**Table 2:** Variables.

Variables	Statistics/level of scales	Possible range of measurements	Time of measurement Women	Time of measurement Newborn/infant	Source of information
Age	Metric variable	In years, women In days/months, newborn/infant 18–45 years, women 0–28 d, newborn 1–6 months, infant	Baseline, t0	Delivery + 28 d, t1 Delivery + 6 months, t2	ID card, woman “U-heft,” newborn/infant U3: 4^th^–5^th^ week U5: 6^th^–7^th^ month
Weight	Metric variable	In kg, woman In kg, newborn/infant	Baseline, t0 Delivery + 28 d, t1 Delivery + 6 months, t2	Delivery + 28 d, t1 Delivery + 6 months, t2	Measurement “U-heft,” newborn/infant U3: 4^th^–5^th^ week U5: 6^th^–7^th^ month
Height	Metric variable	In cm, woman In cm, newborn/infant	Baseline, t0	Delivery + 28 d, t1 Delivery + 6 months, t2	Self-reporting, woman “U-heft,” newborn/infant U3: 4^th^–5^th^week U5: 6^th^–7^th^month
Sex	Nominal variable	Female Male		Delivery + 28 d, t1	“U-heft,” newborn/infant
					U1 delivery
Habitation	Nominal variable	Urban Rural	Baseline, t0 Delivery + 28 d, t1 Delivery + 6 months, t2	Delivery + 28 d, t1 Delivery + 6 months, t2	Self-reporting, woman
Newborn/infant's environment	Nominal variable	Apartment House		Delivery + 28 d, t1 Delivery + 6 months, t2	Self-reporting, woman
Course of delivery	Nominal variable	Vaginal birth Caesarean	Delivery + 28 d, t1		“U-heft,” newborn/infant U1 delivery
Vaginal seeding	Nominal variable	Yes No		Delivery + 28 d, t1	Self-reporting, woman
Newborn/infant nutrition	Nominal variable	Breastfeeding Cow-milk-based infant formula (including hypoallergenic (HA) based formula) feeding or combination		Delivery + 28 d, t1 Delivery + 6 months, t2	Self-reporting, woman
Period of newborn/infant nutrition (supplementary feeding)	Metric variable	From week *x* to week x		Delivery + 28 d, t1 Delivery + 6 months, t2	Self-reporting, woman
Gestational age at birth	Metric variable	In weeks		Delivery + 28 d, t1	“U-heft,” newborn/infant U1 delivery
Dermatological skin assessment, mother	Nominal variable	Coded by ICD11	Baseline, t0 Delivery + 28 d, t1 Delivery + 6 months, t2		Study MD
Dermatological skin assessment, newborn/infant	(i) Eczema (ii) Dry skin (iii) Itchy skin (iv) Rash (v) Flexural dermatitis and/or visible dermatitis (vi) Diaper dermatitis (vii) Hand, mouth, and foot disease	EASI score: (i) Erythema (ii) Edema/papulation (iii) Excoriation (iv) Lichenification EASI score: (i) Severity of signs (1) 0–3 (2) None severe Dry skin: (ii) Desquamation fissures/rhagades (iii) Erythema (iv) Pruritus Itchy skin: (i) If yes: continuous/intermittent Rash: yes/no Diaper dermatitis: yes/no Hand, mouth, and foot disease: yes/no		Delivery + 28 d, t1 Delivery + 6 months, t2	Study MD
Skin care, mother	Open-ended question		Baseline, t0 Delivery + 28 d, t1 Delivery + 6 months, t2		Self-reporting, woman
Skin care, newborn/infant	Open-ended question			Delivery + 28 d, t1 Delivery + 6 months, t2	Self-reporting, woman
Care givers at home	Nominal variable	(i) Father (ii) Sibling (iii) Grandparents (iv) Au pair (v) Nursery nurse (vi) Other		Delivery + 28 d, t1 Delivery + 6 months, t2	Self-reporting, woman
Furred pets at home	Nominal variable	(i) Cat (ii) Dog (iii) Others	Baseline, t0 Delivery + 28 d, t1 Delivery + 6 months, t2		Self-reporting, woman
The atopic predisposition of the mother (at least one parent or sibling with physician-diagnosed AD, asthma, or allergic rhinitis/rhinoconjunctivitis as reported by at least one parent but in otherwise good overall health)	Nominal variable	Yes/no	Baseline, t0		Self-reporting, woman
Antibiotic intake	Nominal variable	Yes/no	Baseline, t0 Delivery + 28 d, t1 Delivery + 6 months, t2		Self-reporting, woman
Alcohol consumption	Nominal variable	Yes/no	Baseline, t0 Delivery + 28 d, t1 Delivery + 6 months, t2		Self-reporting, woman
Skin surface topography roughness: (i) RA (arithmetic mean roughness) in *μ*m (ii) RZ (arithmetic mean roughness from five sampling length) in *μ*m	Metric scaled variable Mean (SD) Median (IQR)	RA: 0.0–100.0 *μ*m RZ: 0.0–100.0 *μ*m	Baseline, t0 Delivery + 28 d, t1 Delivery + 6 months, t2	Delivery + 28 d, t1 Delivery + 6 months, t2	Standardized images via visioscan VC 98 USB (i) RA (arithmetic mean roughness) in *μ*m (ii) RZ (arithmetic mean roughness from five sampling length) in *μ*m Skin area: inner forearm
Epidermal thickness in *μ*m	Metric scaled variable Mean (SD) Median (IQR)	0.0–100.0 *μ*m (40–60 *μ*m)	Baseline, t0 Delivery + 28 d, t1 Delivery + 6 months, t2	Delivery + 28 d, t1 Delivery + 6 months, t2	Standardized images via optical coherence tomography (OCT) Thorlabs' Telesto Epidermal thickness in mm Skin area: inner forearm
Skin stiffness and elasticity total extensibility (Uf, mm)	Metric scaled variable Mean (SD) Median (IQR)	0.00–0.50 Uf in mm/degree	Baseline, t0 Delivery + 28 d, t1 Delivery + 6 months, t2	Delivery + 28 d, t1 Delivery + 6 months, t2	Cutometer MPA 580 total extensibility (Uf, mm), structural elasticity (Ur/Uf) Skin area: inner forearm
Transepidermal water loss (TEWL) Transepidermal water loss through the stratum corneum in *g* per hour per m^2^	Metric scaled variable Mean (SD) Median (IQR)	0.0–60.0 g/m^2^/h	Baseline, t0 Delivery + 28 d, t1 Delivery + 6 months, t2	Delivery + 28 d, t1 Delivery + 6 months, t2	Tewameter 300 Transepidermal water loss through the stratum corneum in *g* per hour per m^2^ Skin area: inner forearm
Skin surface pH	Metric scaled variable Mean (SD) Median (IQR)	>4 or <7.5	Baseline, t0 Delivery + 28 d, t1 Delivery + 6 months, t2	Delivery + 28 d, t1 Delivery + 6 months, t2	Skin pH-meter® PH 905 Concentration of hydrogen ions Skin area: inner forearm
Stratum corneum hydration (SCH)	Metric scaled variable Mean (SD) Median (IQR)	0–120, arbitrary units, whereas higher readings indicate higher stratum corneum hydration	Baseline, t0 Delivery + 28 d, t1 Delivery + 6 months, t2	Delivery + 28 d, t1 Delivery + 6 months, t2	Corneometer CM 825 Capacitance measurement of hydration Arbitrary CM units 0–120 Skin area: inner forearm
Skin microbiome Bacterial diversity Relative abundance of phylotypes (operational taxonomic units, OTUs) Top 5 genera	Metric scaled variable Alpha diversity Shannon index	N number of taxonomic groups, for example, (i) Proteobacteria and Bacteroidetes (ii) Firmicutes (iii) Actinobacteria *N* amount of bacteria (bacterial load)	Baseline, t0 Delivery + 28 d, t1 Delivery + 6 months, t2	Delivery + 28 d, t1 Delivery + 6 months, t2	Skin microbiome Alpha diversity Shannon index Skin area: inner forearm
Hair measurement	Metric variable	Hair thickness Hair density Vellus-terminal-hair ratio	Baseline, t0 Delivery + 28 d, t1 Delivery + 6 months, t2	Delivery + 28 d, t1 Delivery + 6 months, t2	Canfield Hair Metrix average hair width in *μ*m Hair count per cm^2^ Central scalp area, occipital hair area (woman) central hair area (newborn)
Hair colour	Ordinal variable	Hair colour scale, 1–10	Baseline, t0 Delivery + 28 d, t1 Delivery + 6 months, t2	Delivery + 28 d, t1 Delivery + 6 months, t2	Comparison with hair colour scale

## Data Availability

The data used to support the findings of the study are available from the corresponding author upon request.

## Abbreviations

TEWL: Transepidermal water loss
pH: Pondus hydrogenii
SCH: Stratum corneum hydration
EEMCO: Expert group on efficacy measurement of cosmetics and other topical products
OCT: Optical coherence tomography
DNA: Deoxyribonucleic acid
RNA: Ribonucleic acid
SD: Standard deviation
IQR: Interquartile range
AD: Atopic dermatitis
ICH: International conference on harmonization of technical requirements for registration of pharmaceuticals for human use
ICF: Informed consent form
STROBE: Strengthening the reporting of observational studies in epidemiology
